# How storage time affects sensory, chemical, and physical characteristics of flavored olive oil

**DOI:** 10.1002/fsn3.3613

**Published:** 2023-08-11

**Authors:** Tereza Kacalova, Alzbeta Jarosova

**Affiliations:** ^1^ Department of Food Technology Mendel University in Brno Brno Czechia

**Keywords:** chemical analyses, color, flavored olive oil, garlic, rosemary, sensory evaluation, storage

## Abstract

The objectives of this study were to evaluate sensory, chemical, and physical characteristics of olive oil prepared by three flavoring methods and analyze changes during storage (0, 3, and 6 months). Favored olive oil was prepared by three flavoring methods (fresh, dried, and essential oil). Selected sensory, chemical, and color parameters were monitored based on international standards. The color was determined by spectrophotometer. The results confirmed that storage time and flavoring method affect sensory characteristics of the oil. Fresh garlic oil had significantly lower (*p* < .05) fruity smell. The level of pungent taste significantly increased (*p* < .05) in the dried rosemary oil, rosemary essential oil, and fresh and dried garlic flavored oils. The intensity of rosemary smell in the sample flavored with essential oil significantly decreased (*p* < .05) during storage. Opposite effect was observed in the sample flavored with dried rosemary, where the smell significantly increased (*p* < .05) during storage. The intensity of taste significantly increased (*p* < .05) in samples flavored by dried rosemary during storage. The peroxide value of all flavored oils samples increased (*p* < .05) during storage compared to unflavored oil where peroxide value did not change. Color indicators L*, a*, and b* show that addition of fresh rosemary causes the greatest change in olive oil color. The color change, especially the turbidity, was not perceived positive by tasters.

## Graphical abstract

The objectives of this study were to evaluate the changes of sensory, chemical, and physical characteristics of flavored olive oils during storage. The changes during storage time were analyzed to evaluate its influence on the quality of flavored olive oil. Each flavored olive oil was prepared by three flavoring methods and selected sensory, chemical, and color parameters were monitored based on international standards.

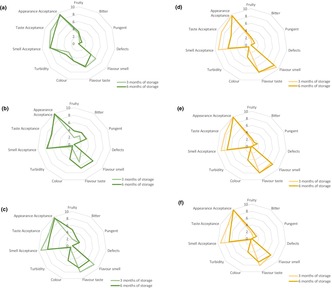

## 1 INTRODUCTION

Olive oil is the main ingredient of the Mediterranean diet and is considered a functional food due to its fatty acid composition, content of polyphenols, and other bioactive compounds. The strategy of enriching food products with bioactive compounds to increase its health benefits is gaining importance worldwide as consumers take greater responsibility for their food choices and own health. Oil flavoring combines two important attributes: great taste and health benefits. The addition of natural sources of biologically active material to oil, such as herbs, spices, olive leaves, pomace, or other compounds from plants and vegetables, form a food product that contributes to the prevention of chronic diseases such as cardiovascular diseases, cancer, or metabolic syndrome (Reboredo‐Rodríguez et al., 2017). A wide range of natural ingredients, such as herbs and spices (chili, garlic, rosemary, basil, mint, oregano, thyme, and lavender), truffles, or fruits (lemon and orange) are used in the process (Abenoza & Sánchez‐Gimeno, 2021). The future of flavored olive oils goes beyond traditional flavoring. An example is walnut, seaweed oil, or olive oil enriched with fish oil (Reboredo‐Rodríguez et al., 2017). Flavored oils, also called “gourmet oil,” are used to dress a wide range of dishes (Gambacorta et al., 2007). Flavored oil is ideal for food pairings, in particular for salads, pasta, seafood, or legumes seasoning (Bahaciu et al., 2018).

The market has gained more interest in flavored oils. Flavored olive oil could increase the use of olive oil among nontraditional customers as their aroma and taste are positively perceived by customers. Their impact on the nutritional value serves as an added value for flavoring with herbs and spices; this is due to their content of natural bioactive compounds (Abenoza & Sánchez‐Gimeno, 2021). Nowadays food marketing of flavoring food has become a main selling point in gaining customers. New flavors are the most frequent path to rejuvenate existing products and expand sales. Flavor is the sensorial attribute that avenues variety and originality. Although flavoring is often used in food products that lack flavor or in which natural flavor is lost, destroyed, or covered (Hui, 2007), the flavoring of olive oil should maintain a balance between the olive oil flavor and the added flavor. This harmony is evaluated in the international flavored olive oil competitions. In this case, the olive oil does not serve as a vector of the flavor but it complements the taste of the product.

Different olive oil flavoring methods can be used: mainly maceration with fresh or dried ingredients, coprocessing during malaxation, addition of essential oil, and microwave‐ or ultrasound‐assisted maceration (Benmoussa et al., 2017; Caponio et al., 2016; Caporaso et al., 2013).

Herbs and spices are a natural source of bioactive ingredients employed to develop a functional flavor‐enriched oil (Harris, 2015). As experienced in previous studies, an improvement in oil stability (peroxide value) occurs when compared to unflavored oils. The incorporation of a flavoring agent has great potential: such as enriching the oil with bioactive compounds, improving sensorial properties, increasing the oxidation stability and shelf life, and modification of the physicochemical properties during storage (Ayadi et al., 2009). The consumption of flavored oil can be likewise beneficial for health as it leads to increase in phenols in the olive oil. In addition, its potential is in added value and market diversification (Abenoza & Sánchez‐Gimeno, 2021). The flavoring process consists of the transfer of bioactive compounds from the flavoring agent into the olive oil which enhances its antimicrobial properties (Bahaciu et al., 2018).

Since some of the bioactive compounds present in the herbs possess a bitter or pungent taste, the final product may have a high intensity of these sensorial attributes. Although bitterness and pungency are positive attributes according to official sensory evaluation of olive oil, their excessive intensity may decrease the acceptance by consumers. Sensory analysis is therefore essential to evaluate the flavored oil characteristics, in particular, flavoring intensity, acceptance, and positive and negative attributes of the olive oil (Reboredo‐Rodríguez et al., 2017).

Herbs and aromatic plants are a natural source of microbial contamination of which the most dangerous microorganisms occurring in flavored oil is *Clostridium botulinum* (Abo et al., 2014). Olive oil provides anaerobic low‐acid environment necessary for the growth and production of its toxin. Visual and organoleptic clues do not provide adequate results about their potential growth (Nummer et al., 2011).

Virgin and extra virgin olive oil are unique among edible oils, they contain small water droplets, where microbes may be present. The physical size of the droplets generally limits their amount. Content of phenols (hydroxytyrosol and tyrosol) can delay the growth of certain bacteria, especially *Streptococcus thermophilus*. Coliform bacteria can survive and reproduce in virgin olive oil containing low levels of phenolic compounds (Ciafardini & Zullo, 2002).

The present study focuses on the investigation of quality characteristics of olive oil flavored with garlic and rosemary. The selected sensorial, chemical, and physical evaluations of oil were conducted in different stages of storage. This study describes the quality of the olive oil flavored with garlic and rosemary using three flavoring methods (maceration of fresh herb, maceration of dried herb, and flavoring by essential oils).

## 2 MATERIALS AND METHODS

### 2.1 Preparation of the flavored olive oil and determination of chemical composition

#### 2.1.1 Sample preparation and process of oil flavoring

The filtered virgin olive oil was obtained from olive fruit (*Olea europea L*.) variety Aayrouni, handpicked from olive orchards in the region of North Lebanon. Aayrouni is an old variety still found in some ancient Lebanese groves. It is mainly cultivated in the North and the Mount Lebanon regions. This cultivar is characterized by high oil content of fruit, ranging from 34% to 37% expressed on fresh weight. This determines their use mainly for oil production (Chehade et al., 2016). A two‐phase continuous extraction system was used. The flavors were selected according to customers’ demand and availability. The rosemary plants (*Salvia rosmarinus*) were bought from a local gardening store (Brno, Czechia). The fresh garlic cloves (*Allium sativum*) were obtained from a Selinger family farm (Lodenice, Czechia). The olive fruits were picked during the harvest of 2019/2020 (Lebanese Genco Olive Oil, Baassir, Lebanon). The concentration of the flavoring material was established according to sensorial evaluation, in particular 7 g of fresh garlic (2 g of dried garlic), 3 g of fresh rosemary (1 g of dried herb), and 75 μL (0.05%) of essential oil (Zan Aromi, Brno, Czechia) were added into 150 mL of oil. A preservation acidification treatment (Abo et al., 2014) for garlic and rosemary was conducted. Unflavored olive oil was prepared as a control group by dividing homogenous unflavored olive oil into transparent jars (volume 150 mL), and samples (*n* = 18) were stored simultaneously with flavored groups of samples. Control samples (*n* = 6) were analyzed at time 0, immediately after sample preparation, then after 3 months of storage (*n* = 6), and after 6 months of storage (*n* = 6). The samples were stored in 150 mL transparent glass bottles in a dark area at room temperature (20 ± 1°C) until analysis.

Analyzed groups of samples:

• Rosemary‐flavored oil:
  ○ RF—oil flavored with fresh rosemary (*n* = 12),
  ○ RD—oil flavored with dried rosemary (*n* = 12),
  ○ REO—oil flavored with rosemary essential oil (*n* = 12).
• Garlic‐flavored oil:
  ○ GF—oil flavored with fresh garlic (*n* = 12),
  ○ GD—oil flavored with dried garlic (*n* = 12),
  ○ GEO—oil flavored with garlic essential oil (*n* = 12).
• Control group:
  ○ N—unflavored oil: control group (*n* = 18)

A total number of 90 samples (*n* = 90) were prepared and analyzed.

#### 2.1.2 Sensory evaluation

The sensorial analyses of the oil were performed immediately after preparation, and then after 3 and 6 months of storage. For each flavor and the time interval, six samples (*n* = 6) were used for sensory analysis. The sensory evaluation was determined by a panel of six (Department of Food Technology, Mendel University in Brno). Panelists were selected from targeted young generation (age 25–30 years old), all Czech nationality. They have attended 1‐year sensory analyses training focusing on sensory analysis methods and passed the exam of receptors´ sensitivity and function (ČSN ISO 8586, ČSN EN ISO/IEC 17025). For the purpose of this study, a 1‐day training focusing on specifics of olive oil tasting was conducted prior to sample evaluation. Informed consent was obtained prior to participation. Six samples per group of oil were served on a white tray to the panelists. Analyses were carried out at the Sensory Laboratory (Department of Food Technology, Mendel University in Brno). For the sample evaluation, 10 mL of oil in transparent glass was served with white bread. The samples were coded with random three‐digit numbers. The following parameters were evaluated: appearance, color, turbidity, smell, taste, and defects on a 10 cm unstructured line scale with both ends anchoring points. Profile sheets were set up for unflavored and flavored oil to range on hedonic scale. Water and white bread were used as neutralizers. Descriptors used are mentioned in Table 1. Positive attributes (fruity, bitter, and pungent) and negative attributes (fusty, musty, rancid, and others) of samples were evaluated (Table 2) based on EU Regulation (2022/2104, 2022/2105). Other descriptors (flavor taste, flavor smell, color, turbidity, and acceptance) were also included (Abenoza & Sánchez‐Gimeno, 2021; Gambacorta et al., 2007). The assessment of color and turbidity was carried out visually.

**TABLE 1:** Descriptors set up for the flavored oil profile sheet.

Descriptor	Anchor point (mm on line scale)	
	0	100
Fruity	Absent	Extremely perceptible
Bitter	Absent	Extremely perceptible
Pungent	Absent	Extremely perceptible
Defects	Absent	Extremely perceptible
Flavor smell	Absent	Extremely perceptible
Flavor taste	Absent	Extremely perceptible
Color	Dark yellow	Dark green
Turbidity	Absent	Extremely perceptible
Appearance—acceptance	Extremely disgusting	Extremely pleasant
Flavor smell—acceptance	Extremely disgusting	Extremely pleasant
Flavor taste—acceptance	Extremely disgusting	Extremely pleasant

**TABLE 2:** Sensory characteristics of unflavored and flavored oils during storage.

Parameter	Method	Storage time			Method	Storage time		
		T0 (*n* = 6)	T3 (*n* = 24)	T6 (*n* = 24)		T0 (*n* = 6)	T3 (*n* = 24)	T6 (*n* = 24)
Fruity	N	2.98 ± 2.00_1_	3.55 ± 2.31^a^_1_	3.98 ± 1.81^a^_1_	N	2.98 ± 2.00_1_	3.55 ± 2.31^a^_1_	3.98 ± 1.81^a^_1_
	RF		3.27 ± 2.16^a^_1_	3.46 ± 2.86^a^_1_	GF		2.58 ± 2.73^a^_1_	2.57 ± 2.80^b^_1_
	RD		2.94 ± 2.53^a^_1_	3.86 ± 2.25^a^_1_	GD		2.20 ± 2.29^a^_1_	2.95 ± 3.13^a, b^_1_
	REO		2.82 ± 2.51^a^_1_	3.99 ± 2.89^a^_1_	GEO		2.60 ± 2.55^a^_1_	3.05 ± 3.11^a,b^_1_
Bitter	N	3.76 ± 2.71_1_	3.05 ± 3.00^a^_1_	3.31 ± 2.77^a^_1_	N	3.76 ± 2.71_1_	3.05 ± 3.00^a^_1_	3.31 ± 2.77^a^_1_
	RF		2.53 ± 3.13^a^_1_	2.73 ± 2.92^a^_1_	GF		1.56 ± 2.08^a^_1_	3.08 ± 3.39^a^_1_
	RD		3.39 ± 2.92^a^_1_	4.14 ± 2.92^a^_1_	GD		2.58 ± 3.07^a^_1_	2.55 ± 3.04^a^_1_
	REO		2.99 ± 2.89^a^_1_	3.44 ± 2.74^a^_1_	GEO		2.36 ± 3.00^a^_1_	2.85 ± 3.15^a^_1_
Pungent	N	3.58 ± 2.74_1_	3.11 ± 2.77^a^_1_	3.45 ± 2.40^a, b^_1_	N	3.58 ± 2.74_1_	3.11 ± 2.77^a^_1_	3.45 ± 2.40^a^_1_
	RF		1.48 ± 2.26^b^_1_	2.41 ± 2.66^a^_1_	GF		1.36 ± 2.37^b^_1_	3.12 ± 3.60^a^_2_
	RD		3.10 ± 3.05^a^_1_	4.28 ± 2.38^b^_2_	GD		1.48 ± 1.94^b^_1_	3.55 ± 3.50^a^_2_
	REO		3.46 ± 3.13^a^_1_	3.43 ± 2.94^a, b^_2_	GEO		2.62 ± 3.04^a, b^_1_	3.26 ± 3.41^a^_1_
Flavor smell	N				N			
	RF		5.54 ± 2.96^a,b^_1_	4.47 ± 2.46^a^_1_	GF		7.84 ± 2.92^a^_1_	8.07 ± 1.81^a^_1_
	RD		3.38 ± 3.06^a^_1_	5.58 ± 2.73^a^_2_	GD		6.79 ± 2.91^a^_1_	8.03 ± 1.22^a^_1_
	REO		7.56 ± 2.73^b^_1_	6.14 ± 2.25^a^_2_	GEO		6.02 ± 3.33^a^_1_	7.56 ± 1.65^a^_1_
Flavor taste	N				N			
	RF		6.34 ± 1.97^a,b^_1_	6.26 ± 1.45^a^_1_	GF		7.31 ± 2.72^a^_1_	7.76 ± 1.14^a^_1_
	RD		5.33 ± 2.45^a^_1_	6.71 ± 1.31^a^_2_	GD		7.16 ± 2.95^a^_1_	7.98 ± 1.35^a^_1_
	REO		7.65 ± 2.05^b^_1_	6.54 ± 1.88^a^_2_	GEO		7.59 ± 2.32^a^_1_	7.25 ± 1.73^a^_1_
Color	N	3.68 ± 2.12_1_	2.20 ± 1.90^a^_2_	3.04 ± 1.99^a^_1_	N	3.68 ± 2.12_1_	2.20 ± 1.90^a^_2_	3.04 ± 1.99^a^_1_
	RF		4.73 ± 2.86^b^_1_	4.48 ± 2.04^b^_1_	GF		2.24 ± 2.07^a^_1_	3.59 ± 1.72^a^_2_
	RD		1.98 ± 1.56^a^_1_	3.80 ± 1.38^a, b^_2_	GD		2.59 ± 2.76^a^_1_	3.66 ± 1.51^a^_2_
	REO		2.14 ± 2.00^a^_1_	3.72 ± 1.79^a, b^_2_	GEO		1.83 ± 1.75^a^_1_	3.75 ± 1.65^a^_2_
Turbidity	N	1.97 ± 0.58_1, 2_	1.97 ± 0.49^a, b^_1_	0.52 ± 0.11^a^_2_	N	1.97 ± 0.58_1, 2_	1.97 ± 0.49^a^_1_	0.52 ± 0.11^a^_2_
	RF		3.59 ± 2.92^a^_1_	2.70 ± 3.77^a^_1_	GF		2.66 ± 3.31^a^_1_	1.24 ± 1.85^a^_1_
	RD		2.63 ± 2.92^a, b^_1_	0.83 ± 1.07^a^_1_	GD		1.63 ± 2.24^a^_1_	0.64 ± 0.79^a^_1_
	REO		1.46 ± 1.96^b^_1_	1.05 ± 1.47^a^_1_	GEO		1.68 ± 2.13^a^_1_	0.72 ± 0.91^a^_1_
Smell acceptance	N				N			
	RF		7.91 ± 1.71^a^_1_	7.66 ± 1.46^a^_1_	GF		8.42 ± 1.57^a^_1_	6.18 ± 2.02^a^_2_
	RD		7.58 ± 1.74^a^_1_	7.33 ± 1.16^a^_1_	GD		8.68 ± 1.26^a^_1_	6.70 ± 2.43^a^_2_
	REO		8.83 ± 1.34^a^_1_	6.74 ± 2.20^a^_2_	GEO		8.82 ± 1.23^a^_1_	6.18 ± 2.79^a^_2_
Taste acceptance	N				N			
	RF		8.04 ± 1.36^a^_1_	7.23 ± 1.17^a^_1_	GF		8.49 ± 1.14^a^_1_	5.66 ± 2.57^a^_2_
	RD		6.57 ± 2.26^a^_1_	6.24 ± 2.27^a^_1_	GD		7.91 ± 1.96^a^_1_	6.42 ± 2.45^a^_2_
	REO		6.99 ± 2.52^a^_1_	7.16 ± 1.46^a^_1_	GEO		7.83 ± 1.85^a^_1_	6.11 ± 2.27^a^_2_
Appearance acceptance	N	8.29 ± 1.55_1_	9.11 ± 0.96^a, b^_1_	8.92 ± 1.41^a^_1_	N	8.29 ± 1.55_1_	9.11 ± 0.96^a^_1_	8.92 ± 1.41^a^_1_
	RF		7.86 ± 2.60^a^_1_	7.81 ± 3.07^a^_1_	GF		9.11 ± 1.48^a, b^_1_	9.11 ± 1.21^a^_1_
	RD		8.49 ± 2.23^a, b^_1_	9.37 ± 0.78^a^_1_	GD		9.68 ± 0.57^b^_1_	9.45 ± 0.67^a^_1_
	REO		9.60 ± 0.57^b^_1_	9.11 ± 1.21^a^_1_	GEO		9.27 ± 1.72^a, b^_1_	9.45 ± 0.66^a^_1_

*Note*: Values are reported as mean ± standard deviation. Different numbers in the same row mean significant difference between evaluated periods (*p* < .05). Different letters in the same column mean significant difference between methods (*p* < .05).

Abbreviations: GD, oil flavored with dried garlic; GEO, oil flavored with garlic essential oil; GF, oil flavored with fresh garlic; RD, oil flavored with dried rosemary; REO, oil flavored with rosemary essential oil; RF, oil flavored with fresh rosemary; N, unflavored oil.

#### 2.1.3 Chemical evaluation

The assessed chemical quality parameters of oil were as follows: free fatty acidity (FFA) and peroxide value (PV). Free fatty acidity was determined by the titration method and expressed as percentage of oleic acid. Analysis was carried out according to the European official methods described in the EU Regulation (2022/2104, 2022/2105).

#### 2.1.4 Physical analyses—color determination CIE lab

Chlorophyll and carotenoid mainly determine the color of oil. The color varies from golden yellow to green. The color measurement was determined using CIEL*a*b* color system. Color was measured at colorimeter Minolta® CM‐3500d (Konica Minolta Sensing Inc., Osaka, Japan) in specular component excluded (SCE) mode, angle 8°, and a slit of 8 mm. Color of oil was measured in 1 cm cuvette. Color was expressed as L* (lightness; black – / white +), b* (yellow + / blue –, attribute), and a* (red + / green –, color attribute) (CIE, 2007). Samples were measured in triplicate, and the mean was used for calculation. Unflavored oil was measured as control group. Total difference in color between samples was calculated for comparison: $\;\Delta E*=\sqrt{\;{\left(\Delta L*\right)}^{2}+{\left(\mathrm{\Delta a}*\right)}^{2}+{\left(\mathrm{\Delta b}*\right)}^{2}\;}$. The value of Δ*E* lower than 1.5 indicates a small difference in color. The values 1.5–3.0 fall into a category of distinct difference while values greater than 3 are classified as very distinct by human eye (Gordillo et al., 2011; Salakova, 2012).

#### 2.1.5 Statistical analysis

In total, 90 samples were analyzed. Data from chemical analyses and color determination were evaluated by one‐way ANOVA with post hoc Tukey's test to determine statistically significant differences. The data of sensory evaluation were tested for normality by Shapiro–Wilk test. Results were not normally distributed and for further comparison, the nonparametric Kruskal–Wallis test was used. A result was considered as statistically significant for *p*‐value less than .05, and the mean comparison, standard deviation, and median were calculated. The Software system (StatSoft Inc.; 2013; STATISTICA (data); version 14; www.statsoft.com) was used for statistical analysis. On the other hand, the statistical evaluation was not performed for ΔE* _ab_ as data were obtained from data reduction of CIE L*, a*, and b* coordinates.

## 3 RESULTS AND DISCUSSION

### 3.1 Chemical evaluation of flavored olive oil

In order to assess the effect of flavoring method, spices, and herbs on quality of olive oil during storage, free fatty acidity (FFA) and peroxide value (PV) were determined. At the beginning of the experiment, the olive oil used for preparation of flavored oils had subsequent characteristics: free fatty acidity 1.09 ± 0.04 (% oleic acid) and peroxide value 8.86 ± 0.63 (meqO_2_/kg of oil). The results of the chemical analysis including free fatty acidity (FFA, % oleic acid) during storage are present in Table 3, and peroxide value (PV, meqO_2_/kg oil) in Table 4. As can be observed, the PV of all analyzed groups did not reach the maximum permitted for their classification as extra virgin oils (20 meqO_2_/kg oil) according to the European regulation for virgin olive oil throughout the storage. The highest PV had oil flavored with fresh rosemary (12.58 meqO_2_/kg oil), the lower PV was observed in fresh garlic‐flavored oil (10.82 ± 0.71) after 6 months of storage. Peroxide value of all flavored olive oils significantly increased (*p* > .05) during storage. The presence of rosemary essential oil in olive oil showed antioxidant activity at early stage of storage as previously studied (Asensio et al., 2013). Previous report demonstrated the protective effect of dried rosemary on olive oil samples after 55 days of storage (Ayadi et al., 2009).

**TABLE 3:** Changes in free fatty acidity (% oleic acid) of flavored olive oils during storage.

Group	After 0 month of storage	After 3 months of storage	After 6 months of storage
	FFA	FFA	FFA
N	1.09 ± 0.04_2_	1.25 ± 0.05^a^_1_	1.03 ± 0.03^a^_2_
RF		1.53 ± 0.05^b^_1_	1.06 ± 0.04^a^_2_
RD		1.25 ± 0.02^a^_1_	0.99 ± 0.01^b^_2_
REO		1.21 ± 0.03^a^_1_	1.01 ± 0.02^ab^_2_
N	1.09 ± 0.04_2_	1.25 ± 0.05^A^_1_	1.03 ± 0.03^A^_2_
GF		1.19 ± 0.02^A^_1_	0.96 ± 0.02^B^_2_
GD		1.21 ± 0.03^A^_1_	0.98 ± 0.01^B^_2_
GEO		1.25 ± 0.03^A^_1_	0.99 ± 0.00^B^_2_

*Note*: Values are reported as mean ± standard deviation. Different numbers in the same row mean significant difference between evaluated periods (*p* < .05). Different letters in the same column mean significant difference between flavoring methods (*p* < .05). Capital letters indicate different flavors.

Abbreviations: GD, oil flavored with dried garlic; GEO, oil flavored with garlic essential oil; GF, oil flavored with fresh garlic; RD, oil flavored with dried rosemary, REO, oil flavored with rosemary essential oil; RF, oil flavored with fresh rosemary; N, unflavored oil.

**TABLE 4:** Changes in peroxide value (meqO_2_/kg oil) of flavored olive oils during storage.

Group	After 0 month of storage	After 3 months of storage	After 6 months of storage
	PV	PV	PV
N	8.86 ± 0.63_**1**_	8.99 ± 0.59^a^_1_	10.58 ± 4.26^a^_1_
RF		7.18 ± 0.15^b^_1_	12.58 ± 0.90^a^_2_
RD		7.44 ± 0.23^b^_1_	11.85 ± 1.27^a^_2_
REO		7.71 ± 0.83^b^_1_	11.21 ± 1.53^a^_2_
N	8.86 ± 0.63_**1**_	8.99 ± 0.59^A^_1_	10.58 ± 4.26^A^_1_
GF		9.03 ± 0.74^A^_1_	10.82 ± 0.71^A^_2_
GD		7.93 ± 0.49^B^_1_	11.31 ± 0.78^A^_2_
GEO		8.12 ± 0.25^AB^_1_	10.90 ± 1.73^A^_2_

*Note*: Values are reported as mean ± standard deviation. Different numbers in the same row mean significant difference between evaluated periods (*p* < .05). Different letters in the same column mean significant difference between flavoring methods (*p* < .05). Capital letters indicate different flavors.

Abbreviations: GD, oil flavored with dried garlic; GEO—oil flavored with garlic essential oil; GF, oil flavored with fresh garlic; RD, oil flavored with dried rosemary; REO, oil flavored with rosemary essential oil; RF, oil flavored with fresh rosemary; N, unflavored oil.

Lipid hydrolases generate free fatty acids which reduce the shelf life of olive oil. This phenomenon is of particular interest in water‐containing lipid matrices, such as virgin olive oil. Free fatty acidity affects sensibility to oxidative degradation of olive oil which leads to the reduction of the shelf‐life (Lozano‐Sánchez et al., 2010). Free fatty acidity of oils flavored by rosemary was lower than the limits set by the EU Regulation 2022/2104 and 2022/2105 for virgin olive oil. The values of samples flavored with essential oil increased during storage (Asensio et al., 2013), however, the data from our study did not confirm this trend which shows the stability of the oil.

### 3.2 Sensory evaluation of flavored olive oil

**FIGURE 1:**
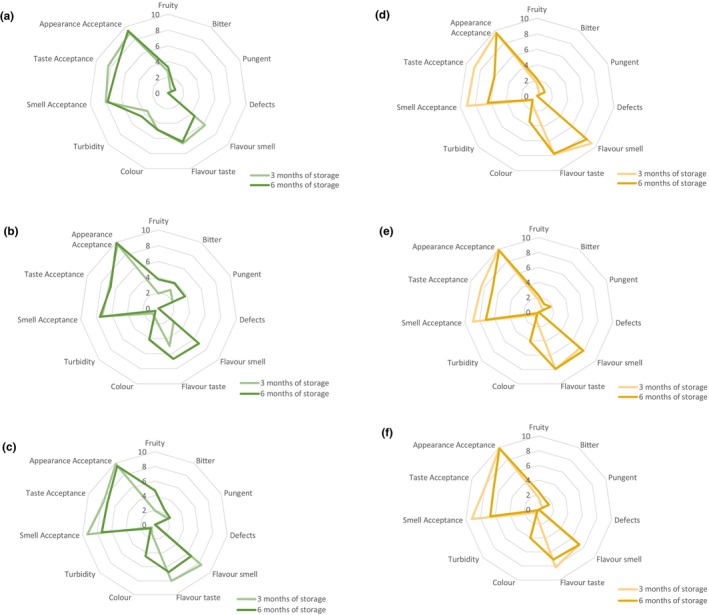
Changes in sensorial attributes in flavored oils during storage. Median (a)—oil flavored with fresh rosemary; (b)—oil flavored with dried rosemary, (c)—oil flavored with rosemary essential oil, (d)—oil flavored with fresh garlic; (e)—oil flavored with dried garlic, and (f)—oil flavored with garlic essential oil.

The flavoring method and storage time affect organoleptic characteristics of oil. The sensory evaluation classifies oil according to the intensity of each attribute. Figure 1 reflects changes in sensorial attributes in flavored oils during storage at radar diagram.

The unflavored oil showed the initial sensory attributes typical of the Aayrouni cultivar: a medium fruity smell (2.98 ± 2.00) with floral notes, and a good balance between bitter and pungent taste (3.75 ± 2.71 and 3.58 ± 2.74, respectively). The fruity smell of unflavored oil did not significantly change (*p* > .05) during storage. The bitter taste of unflavored oil was 3.31 ± 2.77 after 6 months of storage and maintained its level contrary to results of Gambacorta et al. (2007) where loss of bitter and pungent taste was monitored.

After 6 months of storage, the oil flavored with dried rosemary was significantly (*p* < .05) more pungent (4.28 ± 2.38) than oil flavored with fresh rosemary (2.41 ± 2.66). The level of pungent taste significantly increased (*p* < .05) during storage in oil flavored with dried rosemary and oil flavored with rosemary essential oil. Rosemary flavored oil was described as more pungent and bitter by assessors (Abenoza & Sánchez‐Gimeno, 2021).

Bitter taste was more pronounced in oil flavored with dried rosemary (4.14 ± 2.92) although not significant (*p* > .05) as previously reported (Benkhoud et al., 2021). The bitter taste intensity was higher in the oils obtained using infusion method (Caponio et al., 2016).

Oil flavored with dried rosemary (5.58 ± 2.73) and essential oil (6.14 ± 2.25) had significantly (*p* < .05) more intensive rosemary smell than the oil flavored with fresh rosemary (4.47 ± 2.47). However, the intensity significantly (*p* < .05) decreased during storage.

The highest intensity of rosemary taste had oil flavored with dried rosemary (6.71 ± 1.31); its intensity significantly increased (*p* < .05) during storage. An opposite trend was observed in the oil flavored with rosemary essential oil where its intensity significantly decreased (*p* < .05) during storage.

As shown, the difference in color between unflavored oil and oil flavored with fresh rosemary was significant (*p* < .05). The color of oil flavored with fresh rosemary was perceived as greener than unflavored oil. When rosemary was incorporated into oil by maceration, turbidity was shown (Abenoza & Sánchez‐Gimeno, 2021).

Smell acceptance of oil flavored with rosemary essential oil significantly decreased (*p* < .05) during storage; the value after 6 months of storage was 6.74 ± 2.20. The acceptance of smell and taste and the appearance of rosemary‐flavored oil were scored high. According to the results (Akçar & Gümüskesen, 2011), the panelists preferred the rosemary‐flavored oil with higher concentration, particularly 0.07%. Although not significant (*p* > .05), taste of oil flavored by dried rosemary was not preferred. These results were observed also by Benkhoud et al. (2021), where panelists evaluated pungency and irritating smell as negative attributes. Aromatization with fresh rosemary was preferred by consumers (Ayadi et al., 2009).

In comparison with unflavored oil, the oil flavored with fresh garlic had significantly lower value (*p* < .05) of fruity smell after 6 months of storage. The level of pungent taste significantly increased (*p* < .05) during storage in oil flavored with fresh and dried garlic. Decrease in sensorial descriptors such as fruity, bitter, and pungent was detected in the garlic‐flavored oil (Gambacorta et al., 2007).

The panelists showed greater satisfaction with the smell and taste of garlic flavored oils after 3 months of storage, whereas acceptance significantly decreased during storage. Panelists evaluated the appearance of samples with high score while the groups had less variation over time. In general, garlic‐flavored oil achieved better score than rosemary‐flavored oil (Abenoza & Sánchez‐Gimeno, 2021).

All samples were defects free (data not shown) which demonstrates the quality of flavor oils. Positive attributes of flavored and unflavored samples fall into category delicate and medium (El Riachy et al., 2018).

In previous experiments, the sensory notes of the added garlic and rosemary hid the fruity smell and bitter taste. It was suggested to add the flavoring material in smaller quantities. The intensity of smell and taste of flavored oil depends not only on the quantity of flavoring agent but also on the variety, olive ripening index, the quality of flavoring material, and oil extraction process (Baiano et al., 2009, 2016). Caponio et al. (2016) observed that the fruity smell disappeared in garlic‐flavored oil, whereas pungent and bitter taste was present.

### 3.3 Color measurement

In addition to flavoring, polyphenols, chlorophylls, and carotenoids are responsible for the oil color which varies from golden yellow to green tones and plays important role in oxidative stability. Color indicators are described in Table 5, which underwent several changes as a consequence of both flavoring and storage time.

**TABLE 5:** The effect of storage time on color indicators of flavored olive oil.

Parameter	Method	Storage time				Storage time		
		T0 (*n* = 6)	T3 (*n* = 24)	T6 (*n* = 24)	Method	T0 (*n* = 6)	T3 (*n* = 24)	T6 (*n* = 24)
L*	N	88.53 ± 0.34_1_	89.39 ± 0.27^a^_2_	90.07 ± 0.14^a^_3_	N	88.53 ± 0.34_1_	89.39 ± 0.27^A^_2_	90.07 ± 0.14^A^_3_
	RF		84.05 ± 1.17^b^_1_	87.24 ± 0.29^b^_2_	GF		88.68 ± 1.12^B^_1_	91.25 ± 0.08^B^_2_
	RD		89.10 ± 0.26^a^_1_	90.01 ± 0.10^a^_2_	GD		89.59 ± 0.18^A^_1_	90.44 ± 0.06^C^_2_
	REO		89.30 ± 0.19^a^_1_	90.31 ± 0.06^c^_2_	GEO		89.70 ± 0.22^A^_1_	90.33 ± 0.12^D^_1_
a*	N	−2.88 ± 0.07_1_	−3.39 ± 0.11^a^_2_	−3.54 ± 0.39^a^_3_	N	−2.88 ± 0.07_1_	−3.39 ± 0.11^A^_2_	−3.54 ± 0.39^A^_3_
	RF		−2.53 ± 0.38^b^_1_	−3.36 ± 0.12^b^_2_	GF		−3.30 ± 0.35^A^_1_	−4.04 ± 0.05^B^_2_
	RD		−2.91 ± 0.18^c^_1_	−3.17 ± 0.11^c^_2_	GD		−3.47 ± 0.08^A^_1_	−3.59 ± 0.04^C^_2_
	REO		−3.34 ± 0.09^a^_1_	−3.63 ± 0.03^d^_2_	GEO		−3.63 ± 0.05^B^_1_	−3.69 ± 0.03^D^_1_
b*	N	75.13 ± 0.14_1_	73.09 ± 0.09^a^_2_	72.92 ± 0.09^a^_3_	N	75.13 ± 0.14_1_	73.09 ± 0.09^A^_2_	72.92 ± 0.09^A^_3_
	RF		77.22 ± 0.57^b^_1_	77.74 ± 0.36^b^_1_	GF		69.73 ± 0.43^B^_1_	68.50 ± 0.28^B^_2_
	RD		74.64 ± 0.31^c^_1_	75.19 ± 0.48^c^_2_	GD		73.27 ± 0.16^A^_1_	73.05 ± 0.08^A^_2_
	REO		73.39 ± 0.11^d^_1_	72.60 ± 0.07^d^_2_	GEO		72.82 ± 0.19^C^_1_	72.47 ± 0.10^C^_2_

*Note*: Values are reported as mean ± standard deviation. Different numbers (subscript) in the same row mean significant difference between evaluated periods (*p* < .05). Different letters in the same column mean significant difference between methods (*p* < .05). Capital letters indicates different flavors. L* (lightness; black – / white +), b* (yellow + / blue –, attribute), a* (red + / green –, color attribute).

Abbreviations: GD, oil flavored with dried garlic; GEO, oil flavored with garlic essential oil; GF, oil flavored with fresh garlic; RD, oil flavored with dried rosemary; REO, oil flavored with rosemary essential oil; RF, oil flavored with fresh rosemary; N, unflavored oil was the control.

**FIGURE 2:**
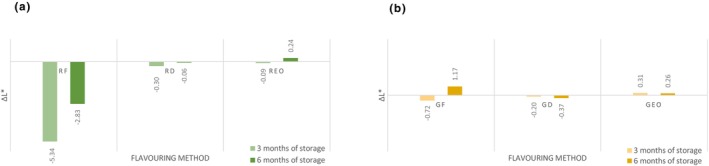
Changes in the lightness (L*) during storage. (a) Rosemary‐flavored oil; (b) Garlic‐flavored oil. ΔL* is compared with control group; ΔL*< 0 sample is darker than control. GD, oil flavored with dried garlic; GEO, oil flavored with garlic essential oil; GF, oil flavored with fresh garlic; RD, oil flavored with dried rosemary; REO, oil flavored with rosemary essential oil; RF, oil flavored with fresh rosemary.

**FIGURE 3:**
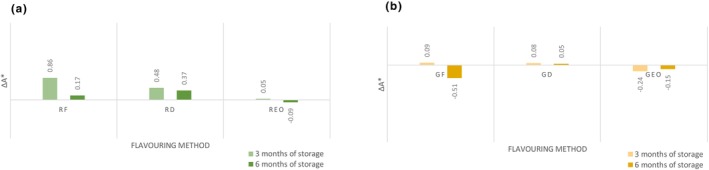
Changes in the color (a*) during storage. (a) Rosemary‐flavored oil; (b) Garlic‐flavored oil. Δa* is compared with control group; Δa*< 0 sample is greener than control. GD, oil flavored with dried garlic; GEO, oil flavored with garlic essential oil; GF, oil flavored with fresh garlic; RD, oil flavored with dried rosemary; REO, oil flavored with rosemary essential oil; RF, oil flavored with fresh rosemary.

**FIGURE 4:**
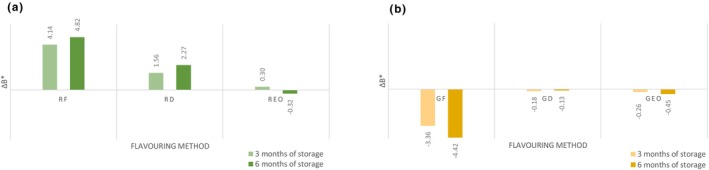
Changes in the color (b*) during storage. (a) Rosemary‐flavored oil; (b) Garlic‐flavored oil. Δb* is compared with control group, Δb* > 0 sample is more yellow than control. GD, oil flavored with dried garlic; GEO, oil flavored with garlic essential oil; GF, oil flavored with fresh garlic; RD, oil flavored with dried rosemary; REO, oil flavored with rosemary essential oil; RF, oil flavored with fresh rosemary.

The lightness (L*) significantly increased (*p* < .05) in flavored and unflavored oil during storage, except oil flavored with garlic essential oil. After 6 months of storage, the L* indicator had the lowest value (87.24 ± 0.29) in samples flavored with fresh rosemary which was significantly lower (*p* < .05) compared to the other groups. The water content present in the fresh rosemary caused a higher turbidity of oil resulting in a darker color. As shown in Figure 2, the values of ΔL* are classified as distinct/very distinct by human eye. Data shown did not confirm the results where dry rosemary oil L* decreased compared to the control group (Ayadi et al., 2009). A significantly lower rating was assigned to the light‐yellow oil color (Gámbaro et al., 2014). The highest value of L* indicator was that of the sample flavored with fresh garlic. The color indicator a* (greenness) shows that the values were significantly increasing (*p* < .05) during storage. The highest value of a* indicator had the sample flavored with dried rosemary (−3.17 ± 0.11): it was significantly (*p* < .05) less green than the unflavored oil. The lowest value of a* indicator was recorded in the sample flavored with fresh garlic (−4.04 ± 0.05), which was significantly (*p* < .05) greener than unflavored control group. Total color changes of a* (Δa*) are shown in Figure 3. Research by Mtimet et al. (2013) showed higher preference for green‐colored oil which is contrary to present sensory analysis. Carotenes and chlorophylls present in oil are responsible for its yellow and green coloration (Gámbaro et al., 2014). Color indicator b* (yellowness) of oil samples significantly changed (*p* < .05) during storage. The highest value of b* indicator after 6 months of storage had sample flavored with fresh rosemary (77.74 ± 0.36), compared to the unflavored sample was significantly (*p* < .05) more yellow. The carotenoid content (β‐carotene, α‐carotene, and lutein) increased in oil macerated with rosemary and significant difference in yellow pigments was observed (Abenoza & Sánchez‐Gimeno, 2021). The minimum b* indicator was recorded in the sample flavored with fresh garlic (68.50 ± 0.28), which was significantly (*p* < .05) less yellow compared to the unflavored sample. Total color changes of b* (Δb*) are shown in Figure 4. The dry garlic powder maceration in oil did not cause significant difference in the content of carotenoids and chlorophyll in comparison with control oil (Abenoza & Sánchez‐Gimeno, 2021); this result was observed in oil flavored with dried garlic after 3 months of storage. Ayadi et al. (2009) did not observe significant difference in a* and b* for all flavored oils.

**FIGURE 5:**
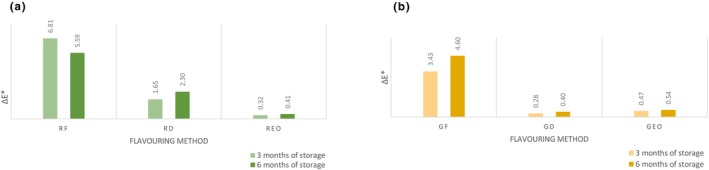
Total color difference ΔE*_ab_. (a) Rosemary‐flavored oil; (b) Garlic‐flavored oil. ΔE*ab is compared with control group. GD, oil flavored with dried garlic; GEO, oil flavored with garlic essential oil; GF, oil flavored with fresh garlic; REO, oil flavored with rosemary essential oil; RD, oil flavored with dried rosemary; RF, oil flavored with fresh rosemary.

A total color change ΔE* _ab_ (Figure 5) after 6 months of storage was very distinct color difference in samples flavored with fresh rosemary (5.6) and fresh garlic (4.6). Oil flavored with dried rosemary had ΔE*_ab_ value 2.3 which falls into category distinct difference. Observed color change could be attributed to the migration of compounds such as pigments, phenolic compounds, organic acids, or essential oils from flavoring agent to oil. Using the fresh material for flavoring has the greatest effect on the color, and the change is associated with higher content of water.

## 4 CONCLUSION

The oil was significantly influenced by the storage time and method of flavoring. The addition of rosemary and garlic enhanced the sensorial characteristics of oil. Sensory evaluation also showed that the descriptor pungent taste undergone greatest change during storage. With regards to profile, the garlic smell and taste of all groups show high stability over time. Chemical analyses showed the increasing trend of peroxide value in flavored oil samples, while free fatty acidity did not increase during storage. Color changes were mostly related to water content of flavoring material. Based on results it can be concluded that appearance of oil flavored with fresh material is less acceptable. Flavoring method did not affect smell and taste acceptance of rosemary‐ and garlic‐flavored oils which is positive for producers. Sensory, chemical, and color analyses bring important information to the customers in making food choices, including purchase. Good sensory quality is likewise required since the taste and smell of these oils need to be accepted by consumers.

## AUTHOR CONTRIBUTIONS

**Tereza Kacalova:** Formal analysis (equal); investigation (equal); validation (equal); visualization (equal); writing – original draft (equal). **Alzbeta Jarosova:** Conceptualization (equal); methodology (equal); supervision (equal); writing – review and editing (equal).

## CONFLICT OF INTEREST STATEMENT

The authors declare that there is no conflict of interest.

## PRACTICAL APPLICATION

This research quantifies and raises awareness of the qualities of olive oil. Provides a tool for perfect olive oil flavoring and pairing. It also predicts consumer preferences and customize the product accordingly.

## Data Availability

The data that support the findings of this study are available on request from the corresponding author.
